# Trimethylamine N-oxide abolishes the chaperone activity of α-casein: an intrinsically disordered protein

**DOI:** 10.1038/s41598-017-06836-2

**Published:** 2017-07-26

**Authors:** Mohd Younus Bhat, Laishram Rajendrakumar Singh, Tanveer Ali Dar

**Affiliations:** 1 Clinical Biochemistry, University of Kashmir, Srinagar, J&K 190006 India; 2 Dr. B. R. Ambedkar Center for Biomedical Research, University of Delhi, Delhi, 110007 India

## Abstract

Osmolytes (small molecules that help in circumventing stresses) are known to promote protein folding and prevent aggregation in the case of globular proteins. However, the effect of such osmolytes on the structure and function of intrinsically disordered proteins (IDPs) has not been clearly understood. Here we have investigated the effect of methylamine osmolytes on α-casein (an IDP present in mammalian milk) and discovered that TMAO (Trimethylamine-N-oxide) but not other methylamines renders α-casein functionless. We observed that the loss of chaperone activity of α-casein in presence of TMAO was due to the induction of an unstable aggregation-prone intermediate. The results indicate that different osmolytes may have different structural and functional consequences on IDPs, and therefore might have clinical implications for a large number of human diseases (e.g., amyloidosis, cancer, diabetes, and neurodegeneration) where IDPs are involved.

## Introduction

Osmolytes are small molecules accumulated by cells to protect from denaturing stresses^1, 2^. These osmolytes protect cells from the hostile stresses by helping to maintain the structural and functional integrity of macromolecules^3–5^. Such stress-protecting osmolytes include polyols and sugars (e.g., glycerol, sorbitol, trehalose, sucrose); amino acids (e.g., proline, glycine) and their derivatives (e.g., taurine, β-alanine); and methyl ammonium compounds (e.g., trimethylamine-N-oxide (TMAO), sarcosine and betaine)^6^. Two defining characteristics of protecting osmolytes are that they stabilize macromolecules against denaturing stresses, and their presence in the cell does not largely alter protein function. Osmolytes are typically accumulated in the intracellular environment up to several millimolar concentrations without causing any cytotoxicity^1–7^. As compared to the other classes of osmolytes, methylamines have been shown to have extraordinary capability to fold denatured or malfolded proteins to native-like species^8–10^, and are commonly found in sharks, elasmobranches, and shallow water invertebrates presumably to keep proteins in their functionally folded states in the presence of high tissue urea levels found in such organisms^11, 12^. Within this class of osmolytes, TMAO is known to be a better stabilizer as compared to other osmolytes including betaine and sarcosine^13, 14^.

Large bodies of data have been generated in the literature about the protein-osmolyte interaction and their thermodynamic effect. Osmolytes are known to modify the protein folding landscape^7^, increase thermodynamic stability^15^, modulate enzyme function^16^, correct temperature sensitive mutants^17, 18^ and help suppress protein aggregation among other things^19^. Almost all of these developments were derived from studies involving globular proteins. In addition to globular proteins, a large portion of the genome of any organism encodes proteins that do not possess a well-defined three-dimensional structure but are involved in various cellular functions. This group of proteins are known as intrinsically disordered proteins (IDPs)^20, 21^. In contrast to the current understanding achieved about the effect of osmolyte on globular proteins, the effect of osmolytes on the structure and function of these IDPs has not been well understood, therefore, it is not known how osmolytes contribute in modulating the biological processes wherein IDPs play a crucial role. In the present study, we have investigated the effect of most potent methylamine osmolytes, TMAO, sarcosine, and betaine on the structure and function of α-casein, an important IDP present in mammalian milk. We demonstrate that TMAO, but not sarcosine or betaine, abolishes the chaperone function of α-casein. Our results indicate that nature of osmolyte may serve as one of the selection criteria for IDPs present in a particular cell type or vice versa.

## Results and Discussion

**Figure 1:**
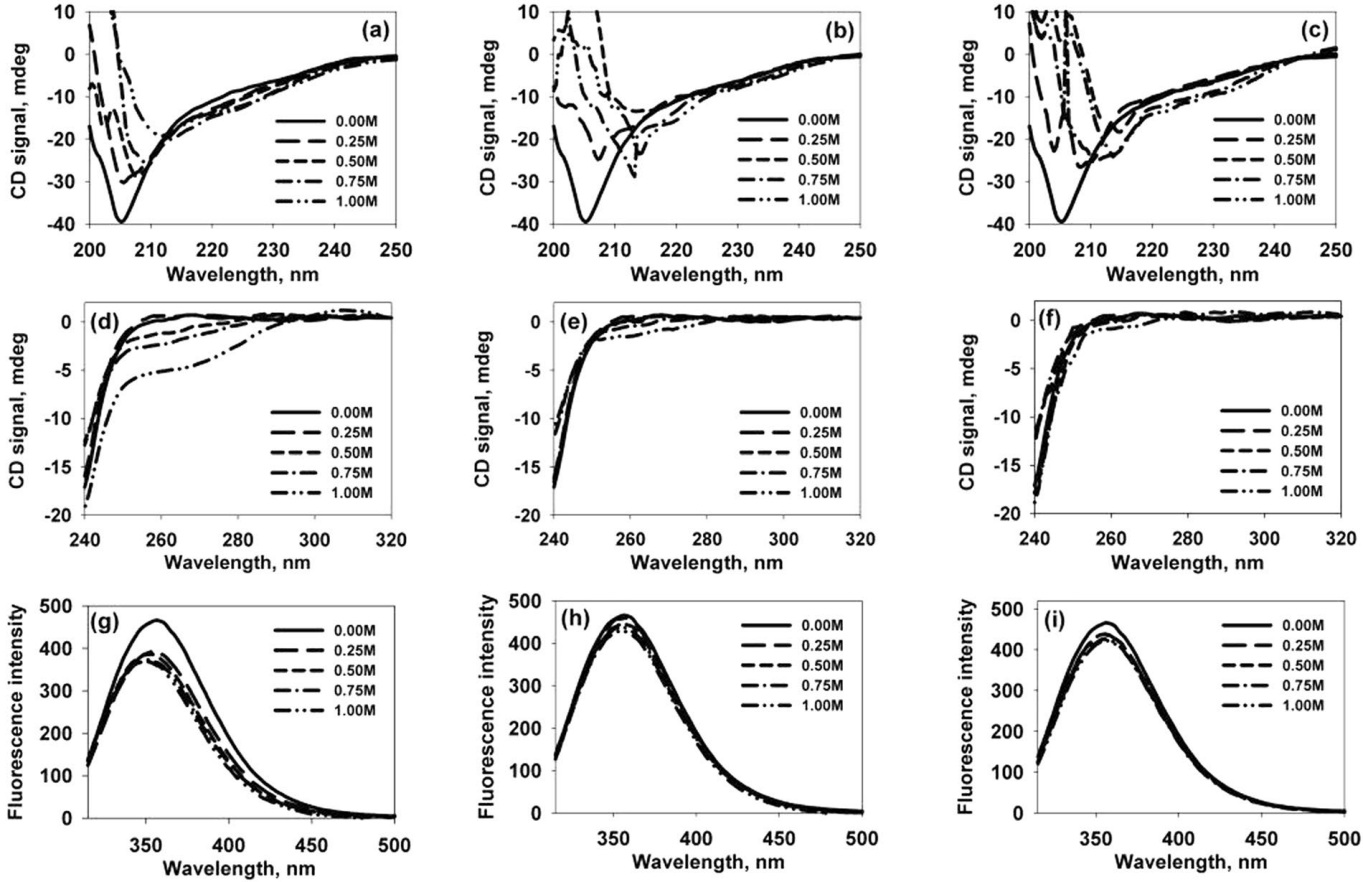
Conformational changes in α-casein by TMAO, sarcosine and betaine: Far- UV CD spectra (**a**,**b**,**c**), near-UV CD spectra (**d**,**e**,**f**) and intrinsic tryptophan fluorescence (**g**,**h**,**i**) of α-casein in presence of TMAO, sarcosine and betaine respectively. Left panel is of TMAO, middle of sarcosine and right one of betaine. Symbols at the right corner denote different concentrations of TMAO, sarcosine and betaine. These data were collected immediately after the sample preparation.

**Figure 2:**
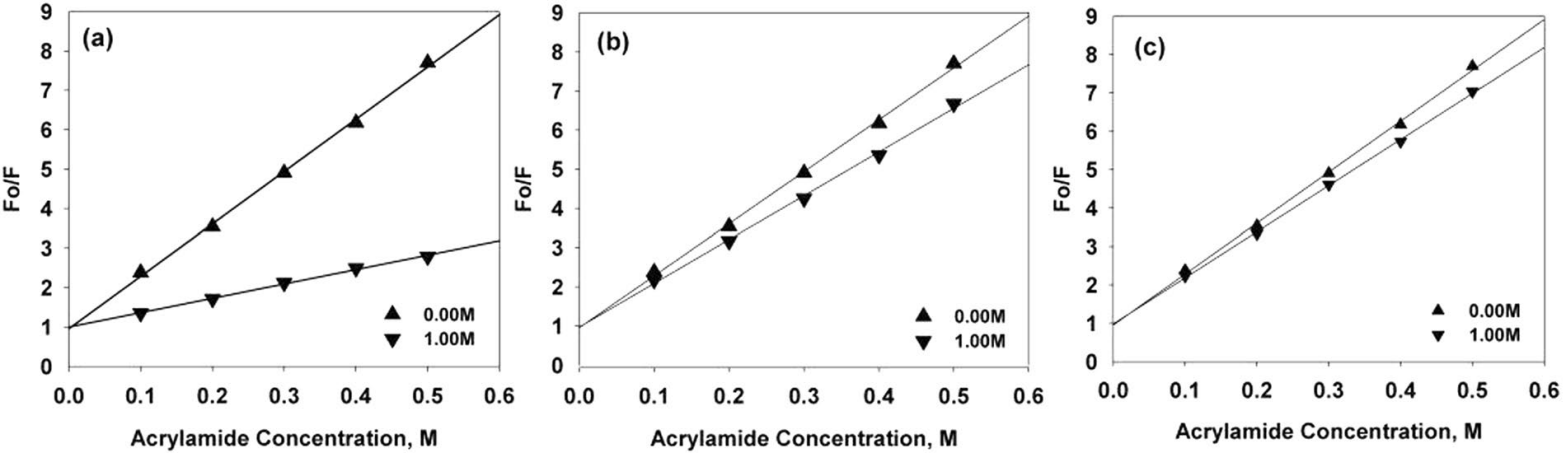
Fluorescence quenching of α-casein. Fluorescence quenching of α-casein in presence of 1 M of TMAO (**a**), sarcosine (**b**) and betaine (**c**). Symbols at the right bottom corner represent concentration of TMAO, sarcosine and betaine. These data were collected immediately after the sample preparation.

The effect of TMAO, sarcosine, and betaine on the structure of α-casein was investigated by measuring the far- and near-UV CD (circular dichroism) spectra in presence of these osmolytes (Fig. 1a–f). We observed that TMAO, sarcosine, and betaine have different effects on the structure of α-casein. Although the secondary structure was not significantly affected by any of the osmolytes, as indicated by the very small change in the θ_222_ region, TMAO induced a significant increase in the tertiary structure (as evidenced from the increased in the 275 nm region) while the effect of sarcosine and betaine was minimal or insignificant. The tertiary structure alterations were again confirmed by tryptophan fluorescence measurements. It was observed that there was a prominent blue shift in case of α-casein incubated with TMAO (Fig. 1g) while there was no blue shift and hypochromicity in case of sarcosine and betaine (Fig. 1h,i). No observable increase in secondary structure, but a considerable increase in the tertiary structure of α-casein by TMAO indicates compaction of α-casein rather than structure creation. To exclude structure creation and verify the compaction, α-casein should have an altered exposure pattern of some of the tryptophan residues in the protein. To test this, we analyzed the quenching behavior of tryptophan by acrylamide (Fig. 2). The calculated Stern-Volmer constants, Ksv of 13.18 for control α-casein, and 3.64, 11.16, and 11.98 in the presence of 1 M each of TMAO, sarcosine, and betaine respectively revealed that TMAO, but not sarcosine or betaine, shows a large reduction in quenching by acrylamide. Prevention of quenching by acrylamide in TMAO treated α-casein indicates that many of the tryptophan residues have been buried or shifted to a more non-polar environment due to the compaction. As expected, neither sarcosine nor betaine exhibit a significant reduction of the quenching behavior due to no major alteration in the native state structure of α-casein. Consistent with our results on sarcosine and betaine, it has been reported that some osmolytes or crowding agents, do not influence the disorderness of an IDP^22^. On the other hand, TMAO and trehalose have been shown to induce compaction or structure formation in the intrinsically disordered N-terminal domain of glucocorticoid receptor^23^. Taken together the results led us to conclude that different osmolytes, even within the same class, have different structural consequences on IDPs.

**Figure 3:**
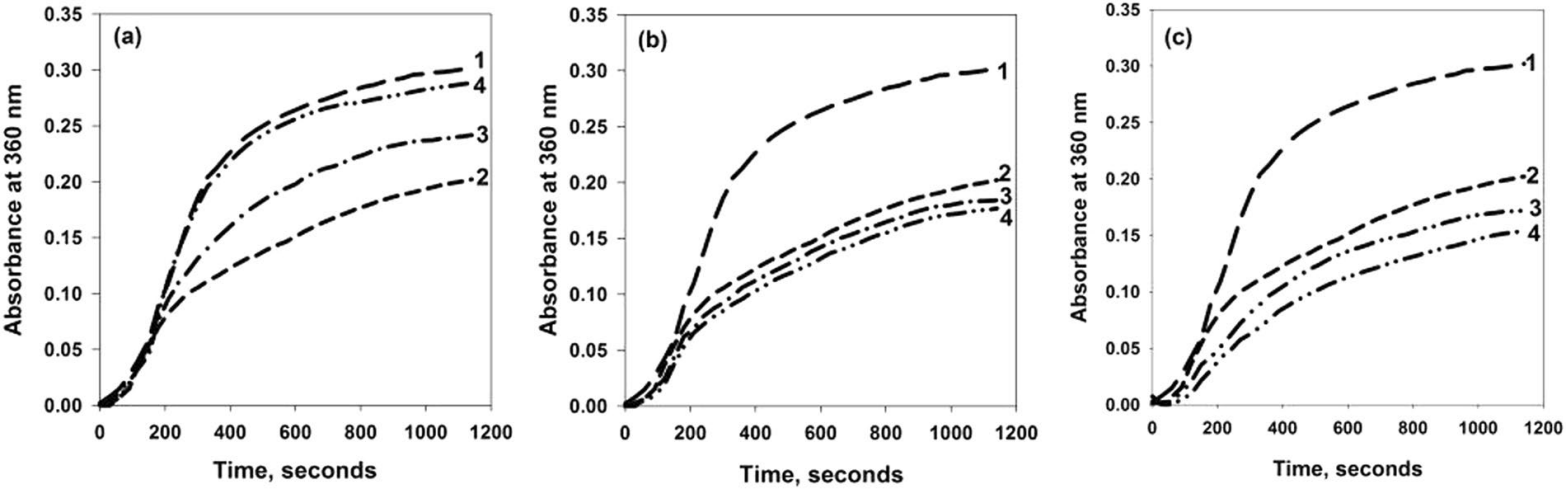
Effect of TMAO, sarcosine, and betaine on the molecular chaperone activity of α-casein: Activity measurement of α-casein in presence of TMAO (**a**), sarcosine (**b**) and betaine (**c**) by light scattering at 360 nm. Curve 1 (catalase control), curve 2 (catalase in presence of α-casein, unincubated with osmolyte), curve 3 (catalase in presence of α-casein, briefly incubated with 1 M TMAO, sarcosine or betaine for less than 30 seconds), curve 4 (catalase in presence of α-casein, incubated overnight with 1 M TMAO, sarcosine or betaine). TMAO, sarcosine and betaine alone do not influence aggregation behavior of catalase, data not shown. Each aggregation kinetics of catalase (curves 1–4) was initiated upon immediate addition of α-casein.

**Figure 4:**
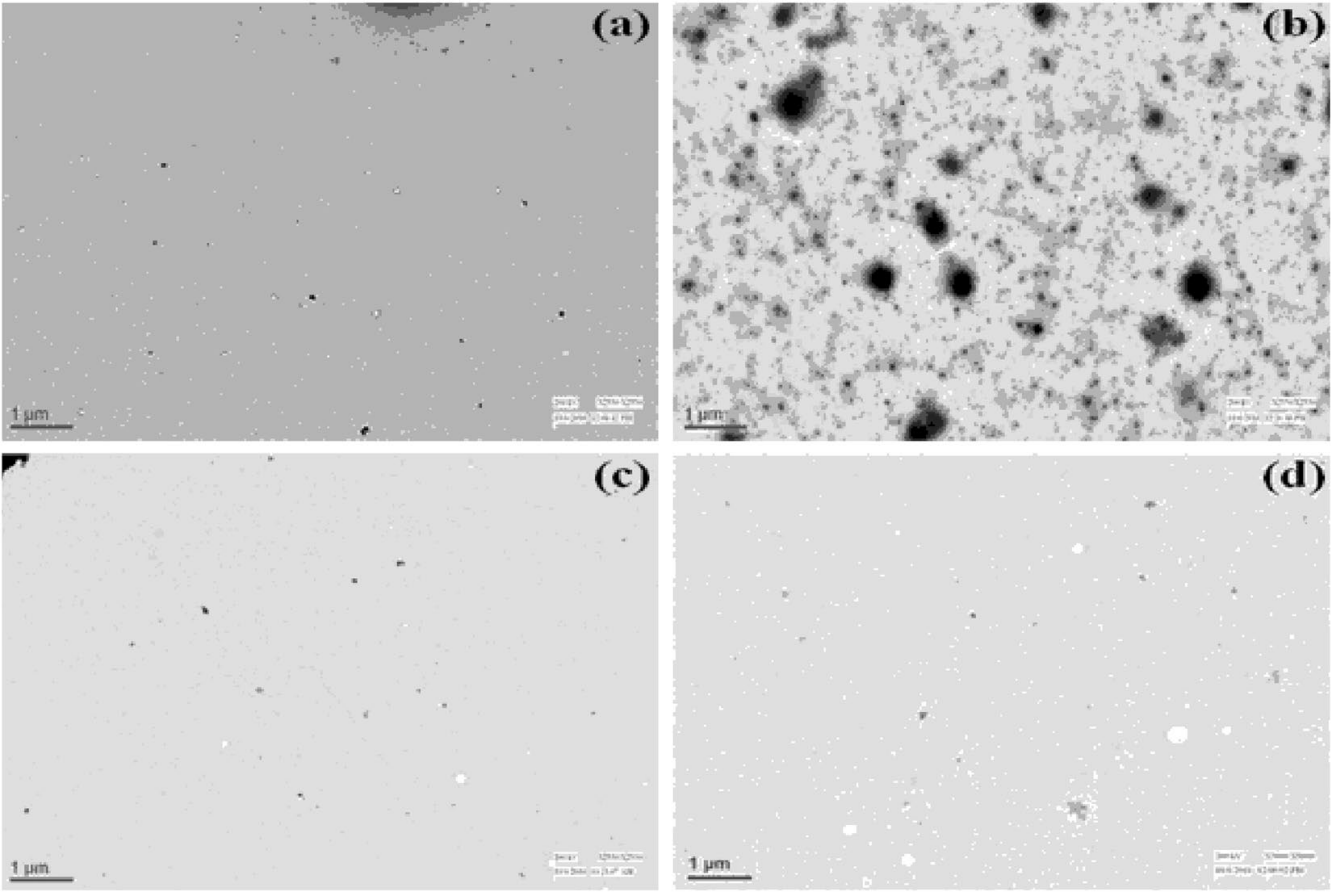
TEM (transmission electron microscopy) of α-casein in presence of TMAO, sarcosine, and betaine. α-casein incubated overnight in the absence (**a**) and the presence of TMAO 1 M (**b**), sarcosine 1 M (**c**) and betaine 1 M (**d**). The scale bar is 1 µm. Data was collected 16 hour after sample preparation.

**Figure 5:**
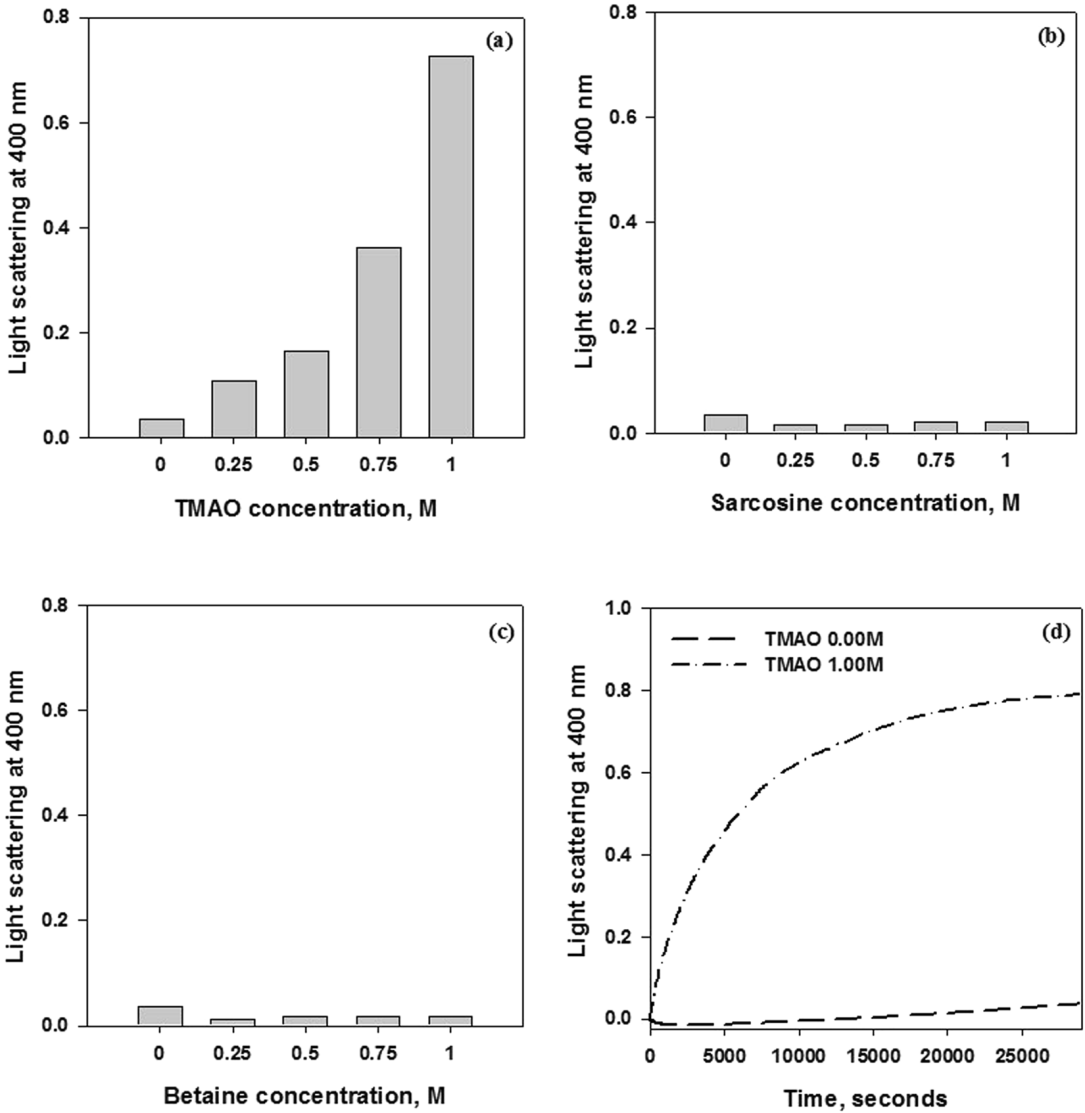
Light scattering of α-casein. Light scattering by α-casein at 400 nm in presence of varying concentrations of TMAO (**a**), sarcosine (**b**) and betaine (**c**). Data were collected 16 hours after sample preparation. Time-dependent aggregation kinetics of α-casein in absence and presence of 1 M TMAO from 0–8 Hours (**d**).

α-casein has been known to possess molecular chaperone activity and help in the protection of aggregation-prone proteins in milk^24, 25^. The differential effects of methylamine osmolytes on the structure of α-casein again prompted us to examine the activity of α-casein in the presence of TMAO, sarcosine, and betaine. For this, we have investigated the ability of TMAO-treated, sarcosine-treated or betaine-treated α-casein sample against catalase aggregation by measuring the light scattering intensity at 360 nm against time (Fig. 3). We observed that TMAO-treated sample reduced chaperone activity of α-casein (Fig. 3a, curve 3) while the chaperone activity of α-casein in presence of either sarcosine or betaine (curve 3 of Fig. 3b and c) was higher than the untreated control (curve 2, Fig. 3). The results indicate that TMAO has a different effect than either sarcosine or betaine on α-casein activity. The degree of disorder is an important intrinsic attribute for any IDP to have its proper functional activity^26, 27^. Therefore, we reasoned that compaction of α-casein by TMAO alters the degree of disorder of α-casein, ultimately resulting in the lowering of its chaperone function. Interestingly, when we incubated the samples for longer time periods (16 hours), there was complete loss of α-casein activity in case of TMAO treated α-casein (curve 4 of Fig. 3a) where as sarcosine- and betaine-treated α-casein activity was relatively unaltered (curve 4 of Fig. 3b and c). Taken together, these results indicate that TMAO-induced compact structure might be an unstable, aggregation-prone kinetic intermediate that ends up by converting to aggregates. To further look at the possibilities we have analyzed the aggregation propensity of α-casein at longer time period (16 hours) by measuring the light scattering as well as electron micrograph. It is evident in Figs 4 and 5 that there is an appearance of aggregates in the case of TMAO treated α-casein whereas, in case of sarcosine or betaine, there was no significant difference between the treated and untreated α-casein samples at this longer time period. We, therefore conclude that TMAO induces an unstable kinetic (aggregation prone) intermediate whereas sarcosine and betaine do not exhibit such kind of intermediate formation due to insignificant alteration of α-casein structure vis-à-vis function. We also investigated the time point for the initiation of α-casein aggregation in absence and presence of TMAO by measuring the time-dependent light scattering (Fig. 5d). We observed that α-casein initiates oligomerization as soon as TMAO is added, confirming that the TMAO-induced compact structures are rapidly oligomerizing conformers.

**Figure 6:**
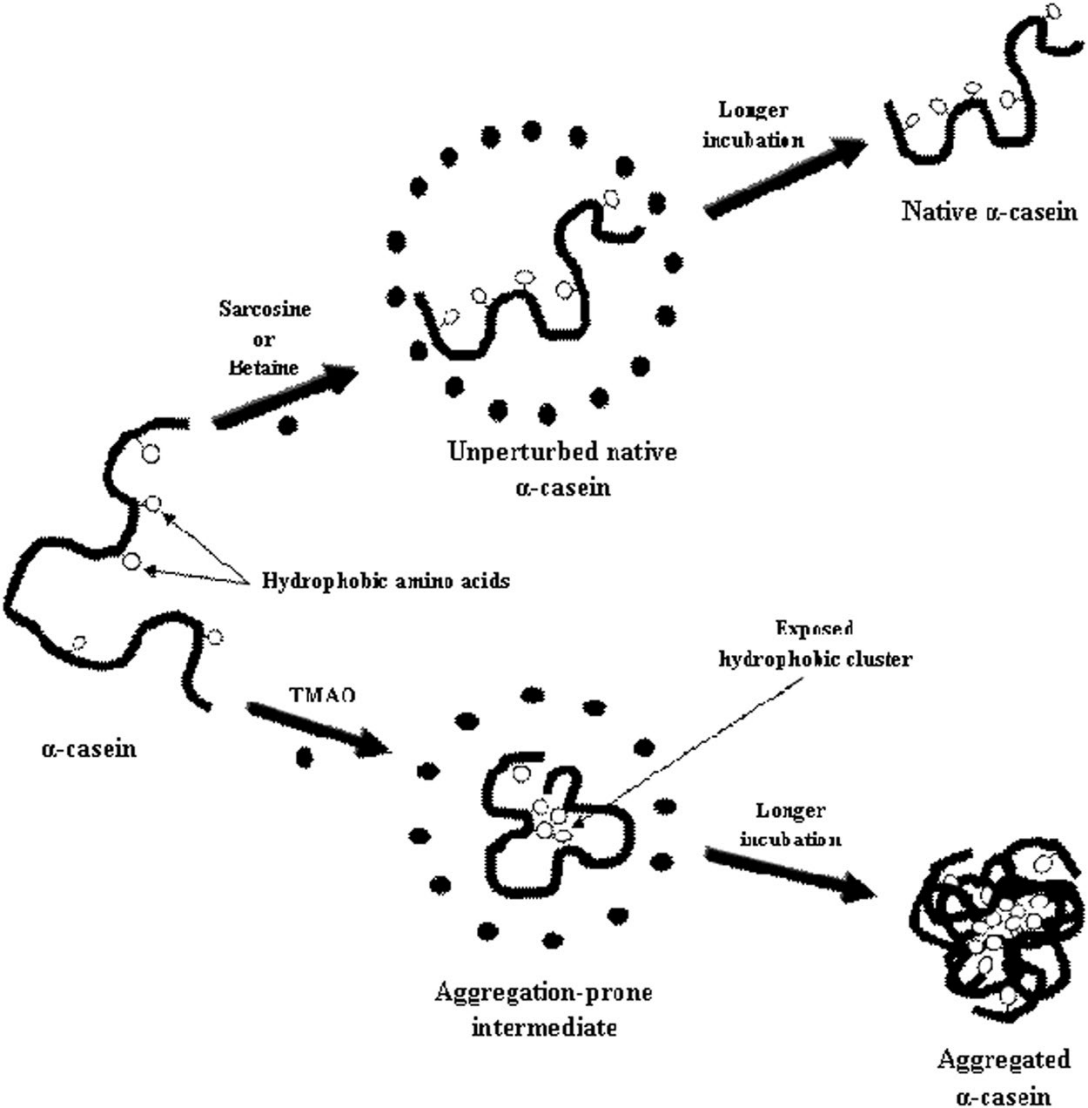
Schematic representation of mechanism of methylamine osmolytes on intrinsically disordered protein, α-casein.

**Figure 7:**
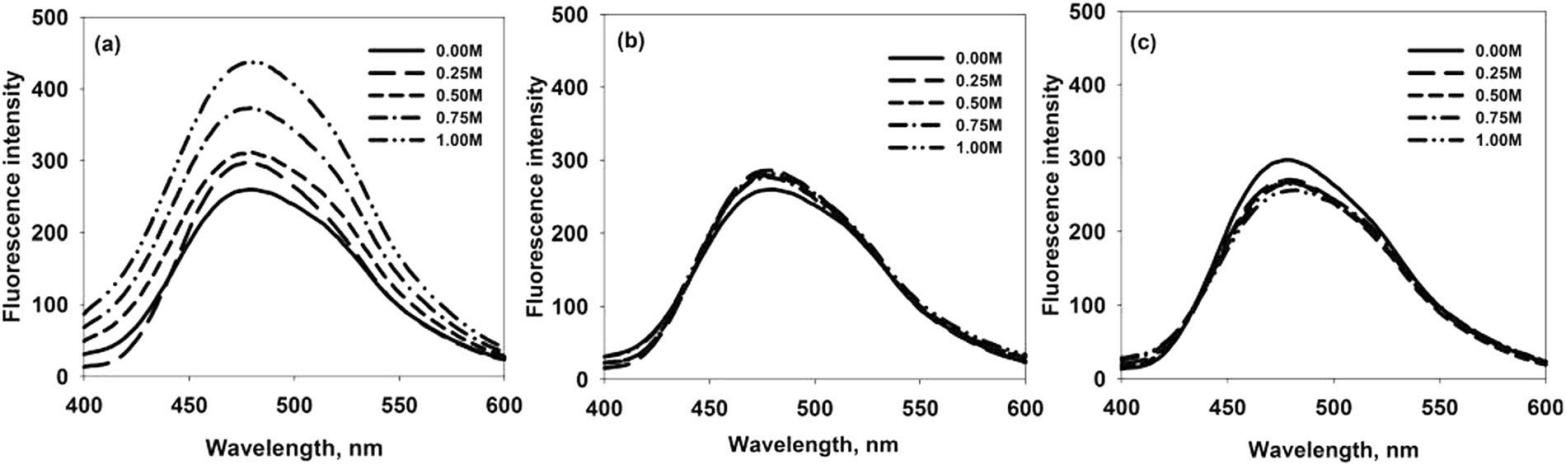
ANS fluorescence spectra of α-casein in presence of TMAO, sarcosine, and betaine. ANS fluorescence in presence of TMAO (**a**), sarcosine (**b**) and betaine (**c**). Symbols at the right upper corner denote different concentrations of TMAO, sarcosine and betaine. These data were collected immediately after the sample preparation.

Methylamines are well-known for their ability to induce protein folding^28, 29^. Our results indicate that ability to induce folding of an IDP is an intrinsic property of the osmolyte. In our case TMAO induces partial folding of α-casein whereas sarcosine and betaine does not show such effect (as depicted in Fig. 6). Since, IDPs do not intrinsically collapse to a globular conformation due to the presence of less number of hydrophobic residues and high number of charged amino acids, the folded intermediate undergoes partial hydrophobic collapse wherein it competes for its intrinsic disorder, and at the same time experiences the folding pressure due to clustering of hydrophobic residues in presence of osmotic stress. During this process, exposed hydrophobic clusters interact with each other leading to the initiation of aggregation. To examine for the existence of such hydrophobic clusters (that promote aggregation), we performed ANS (1-anilinonapthalene-8-sulfonic acid) binding experiments of the structures formed by TMAO, sarcosine, and betaine respectively (Fig. 7). ANS has been known to specifically bind to the exposed hydrophobic clusters which are exposed to the solvent^30^. As expected there was an apparent binding of ANS (as revealed by a large increase in relative fluorescence intensity at λ_max_) to the TMAO-induced structure but not the sarcosine- or betaine-induced structure. Taken together the results indicate that TMAO induces partial folding of α-casein but not sarcosine and betaine.

On the molecular level, osmolyte-induced protein folding is explained by the osmophobic effect, which is the predominant unfavorable interaction that osmolytes have with the peptide backbone. Interaction with the side chains does not contribute significantly^31, 32^. It appears that the osmophobic effect exerted by TMAO on α-casein is large enough to induce partial folding of the disordered protein while the effects of sarcosine or betaine are not significant enough to induce folding. In agreement with this argument, previous transfer free energy (Δg_tr_) measurements have clearly demonstrated that transfer free energy of peptide backbone from water to 1 M TMAO solution is quite higher than that of sarcosine and betaine solutions^31, 33^. Although all osmolytes used in this study are methylamines, sarcosine and betaine are glycine derivatives (mono-methyl glycine and tri-methyl glycine respectively) while TMAO is not. Additionally, sarcosine and betaine have carboxylate as functional group while TMAO has amine oxide. The rationality of osmophobic effect involving peptide backbone in osmolyte solution follows from consideration of hydrogen bonding capabilities of the two competing solvent components, water and osmolytes. The differences in the molecular structure might lead to stronger preference for hydrogen bonding between osmolyte and water (in case of sarcosine and betaine) while lower preference in case of TMAO and water, thereby making water-water hydrogen bonding stronger in presence of TMAO. This scenario allows for a stronger preferential hydration (unfavorable interaction) between peptide backbone and TMAO in relation to the other osmolytes, consistent with the transfer free energy data. Interestingly, a recent MD simulation study revealed that TMAO and betaine interact differently with proteins by virtue of having different hydrogen bonding pattern with water molecules^34^. It has been reported earlier that disorderness prevails under crowded conditions^22^. Our study further confirms that the nature of the osmolyte or the magnitude of the osmophobic effect determines the disorderness of an IDP under osmolyte-induced crowded conditions.

Our findings provide significant evidence that TMAO but not other methylamines render α-casein functionless. Mechanistically, the ability of TMAO to suppress the chaperone activity of α-casein is due to the formation of kinetically unstable aggregation-prone intermediate. The findings indicate that different osmolytes, even if they belong to the same class, have different structural and functional consequences on IDPs. Since aggregation of IDPs is the common hallmark of various human diseases (e.g., amyloidosis, neurodegeneration, cancer and diabetes) presence of certain types of osmolytes may help to potentiate the disease pathology.

## Experimental Procedure

### Materials

Commercially lyophilized protein bovine α-casein, Trimethylamine-N-oxide (TMAO), sarcosine, betaine, sodium cacodylate trihydrate, ANS and acrylamide were purchased from Sigma-Aldrich Chemical Company USA. Potassium chloride was purchased from Merck India. All the chemicals which are of analytical grade were used without further purification. Bovine α-casein was extensively dialyzed overnight at 4 °C against 0.1 M KCl, pH 7.4. Stock solution of protein was filtered using 0.22 µm syringe filters. The concentration of α-casein was determined by Jasco V-660 spectrophotometer, using molar absorbance coefficient (ε) values of 11,000 M^−1^cm^−1^ at 280 nm. All the experiments were carried out in 0.05 M cacodylate buffer (pH 7.4) at 37 °C. All optical measurements were taken in appropriate degassed buffer.

### Experimental methods

#### Circular Dichroism measurements

CD measurements in triplicates were collected in Jasco J-810 spectropolarimeter using quartz cuvettes of 0.1 and 1.0 cm path lengths for far- (200–250) and near-UV (240–320) region at 37 °C. Protein concentration used for both far- and near- UV CD was 0.35 mg/ml. The scan rate was 100 nm/min and each scan was an average of 3 accumulations. All the spectra were corrected for appropriate buffer contributions.

#### Fluorescence measurements

Fluorescence emission spectra (in triplicates) were measured in Perkin Elmer LS 55 spectrofluorimeter in a 3 mm quartz cuvette at an excitation wavelength of 295 nm from 300–500 nm at 37 °C. Protein concentration was 5 µm with slit widths for both excitation and emission set at 10 nm.

Fluorescence quenching experiments with acrylamide were also performed under conditions similar to fluorescence emission spectra described above. Samples were titrated with increasing concentrations of acrylamide from 0.1 M–0.5 M. Data was fitted using Stern-Volmer equation F_o_/F = 1 + K_sv_ [Q], where Q is the concentration of quencher used, F_o_ is fluorescence without quencher and F is fluorescence in presence of varying concentration of quencher.

For ANS binding experiments, fluorescence emission spectra were recorded in the region of 400–600 nm and the excitation wavelength was set at 350 nm. ANS concentration was 16 times higher as that of protein concentration. All the samples were incubated for 30 minutes after adding ANS. Each spectrum was recorded at least 3 times and necessary blanks were subtracted.

#### Transmission electron microscopy

Samples of α-casein in absence and presence of 1 M each of TMAO, sarcosine and betaine were taken on a copper grid (100 mesh × 250 µm pitch) and left at room temperature for 5 minutes. Uranyl acetate solution (1.0%) was added to samples present on the copper grid to negatively stain them. Samples were left to air dry before getting examined using FEI Tecnai G2-200kV HRTA transmission electron microscope operating at 200 kV.

#### Activity measurements

Chaperone activity of α-casein against amorphously aggregating catalase with heat stress at 55 °C was determined by light scattering at 360 nm using Jasco V-660 spectrophotometer in 10mm quartz cuvette. Catalase and α-casein were taken in the ratio of 1:1. Catalase was taken at a concentration of 0.375 mg/ml.

#### Light scattering measurements

Light scattering measurements of α-casein at 400 nm were taken using Jasco V-660 spectrophotometer in absence and presence of various concentrations of TMAO, sarcosine or betaine. Protein concentration used was 0.5 mg/ml. All the measurements were taken at 37 °C and were recorded in triplicates.

Using the same procedure, time dependent aggregation kinetics of α-casein was also measured in absence and presence of 1 M TMAO from 0–8 hours.
